# A Prospective Clinical Cohort Investigation on Zirconia Implants: 5-Year Results

**DOI:** 10.3390/jcm9082585

**Published:** 2020-08-10

**Authors:** Ralf-Joachim Kohal, Benedikt Christopher Spies, Kirstin Vach, Marc Balmer, Stefano Pieralli

**Affiliations:** 1 Department of Prosthetic Dentistry, Center for Dental Medicine, Faculty of Medicine, Medical Center, University of Freiburg, 79106 Freiburg, Germany; benedikt.spies@uniklinik-freiburg.de (B.C.S.); stefano.pieralli@uniklinik-freiburg.de (S.P.); 2 Institute of Medical Biometry and Statistics, Faculty of Medicine and Medical Center, University of Freiburg, 79106 Freiburg, Germany; kv@imbi.uni-freiburg.de; 3 Clinic of Reconstructive Dentistry, Center of Dental Medicine, University of Zurich, 8032 Zurich, Switzerland; Marc.Balmer@zzm.uzh.ch

**Keywords:** dental implants, zirconium oxide, clinical study, survival rate, alveolar bone loss, patient satisfaction

## Abstract

Mid-term data on zirconia oral implants is very rare. Therefore, the aim of this prospective clinical investigation was to evaluate the survival rate and the marginal bone loss of a one-piece zirconia implant after five years. Patient-reported outcomes were also recorded. Zirconia implants to support single crowns (SC) or a 3-unit fixed dental prosthesis (FDP) were placed and subsequently restored. After the insertion of the implants, at prosthetic delivery, and after five years, standardized radiographs were taken to evaluate marginal bone loss (MBL). For bone tissue evaluation, linear mixed models with random intercepts were fitted. Twenty-seven patients received one implant for an SC and 13 patients received two implants for a 3-unit FDP. Three patients each lost one implant for an SC before prosthetic delivery. Thirty-five patients were seen after five years, and no further implant was lost. The cumulative five-year implant survival rate was 94.3%. The MBL from implant installation up to five years was 0.81 mm. The MBL from implant installation to prosthetic delivery was statistically significant (*p* < 0.001). Patients perceived a significant improvement in function, esthetics, sense, speech, and self-esteem from pretreatment up to the five-year follow-up. The present findings substantiate the clinical applicability of this implant system.

## 1. Introduction

Zirconia oral implants are regarded as an addendum to the present implant armamentarium by clinicians, while implants made of titanium are considered the gold standard. However, it is expected that the share of these implants will increase in the near future [1]. Currently available clinical data on zirconia implants are limited to fixed prosthetic rehabilitations (especially single crowns (SCs) and short-span fixed dental prosthesis (FDPs)) and show high short-term survival rates (98% after 1 year and 97% after 2 years), which are comparable to two-piece titanium implants [2,3,4,5]. Medium and long-term clinical data on zirconia implants are still scarce in the scientific literature [6,7,8]. Zirconia as a metal-free alternative to titanium for the manufacturing of implants is of interest for our patients, since this material can positively influence the esthetic outcome of a reconstruction due to the tooth-like color [9,10]. A second potential advantage might be seen in lacking metallic particles, which are liable in causing adverse effects such as hypersensitivity. However, the latter argument presents a topic of controversial discussion in the dental implant community [11,12,13]. Additionally, some investigations showed that (zirconia) ceramics are less prone to bacterial attachment/peri-implant infection [14,15,16,17]. A further argument in favor of zirconia as a ceramic implant material is the desire of certain patients to be restored without metals.

Zirconia implants present good mechanical characteristics which are attributed to the crystalline structure, namely yttria-stabilized tetragonal zirconia polycrystal (Y-TZP) [18,19]. Implant design (1-piece versus 2-piece) and bulk material (Y-TZP versus zirconia composites) showed to have an influence on their mechanical behavior. Regarding implant stability in preclinical situations, a systematic review and meta-analysis by Bethke et al. [20] summarized the results of different investigations. A mean bending moment at fracture of approximately 390 Ncm was calculated in that study. Regarding the implant design, one-piece zirconia implants (431 Ncm) were significantly more stable than the two-piece implants (291 Ncm). Bethke et al. mentioned that “one might consider a minimum fracture resistance of 200 Ncm sufficient to guarantee clinical safety” after taking into account the highest (clinical) bending moment of 95 Ncm including a safety buffer of 100%.

To further improve the physical characteristics of zirconia ceramics by reducing the low thermal degradation (LTD) phenomenon [21], a zirconia-based composite material was created by adding 20 wt% of alumina to the zirconia material named alumina-toughened zirconia (ATZ) [22,23]. ATZ implants showed comparable osseointegration rates to Y-TZP or titanium implants in vivo [24]. The bending moment to fracture of the ATZ implants was reported to be between 274 and 381 Ncm [23]. Furthermore, initial data from short-term prospective clinical investigations are promising [25,26]. The aim of clinical science is to provide long-term clinical data to confirm preclinical and short-term findings. Therefore, the present prospective cohort investigation evaluated the clinical outcome of an ATZ ceramic implant after 5 years of follow-up. The primary aim of this clinical trial was to assess the survival rate of this implant system. Further aims were the analysis of peri-implant marginal bone loss (MBL), soft tissue health, as well as patient-reported outcome measures (PROMs).

## 2. Materials and Methods

### 2.1. Null Hypothesis

The null hypothesis assumed there is no difference in terms of implant survival rates between one and five years after implant placement.

### 2.2. Design of the Investigation

The present investigation was designed as a monocenter clinical prospective observational cohort study. The approval to conduct the trial was obtained from the participants, and their rights have been protected by the ethics committee of the Medical Center—University of Freiburg, Germany (investigation number: 354/07; Ethics committee votum: 22 February 2008). A total of 40 male and female patients between the age of 18 and 70 years were recruited from patients of the Department of Prosthetic Dentistry on a convenience basis. They had to be in need of an implant-supported SC or a three-unit implant-supported FDP with terminal abutments. They had to provide good oral hygiene, be compliant, and present as systemically healthy for study inclusion. A stable occlusal relationship with no signs of pronounced bruxism and sufficient bone volume to receive at least implants with a size of 3 × 9 mm and a primary stability of at least 30 Ncm were considered as further inclusion criteria. Reasons for exclusion included pregnancy, a history of alcohol/drug abuse, or health conditions, which did not permit the surgical procedure. Local contraindications comprised the necessity of larger augmentative procedures before implant installation to obtain a prosthetically correct implant position transversally and impairments of the implant site because of tumors, irradiation, or chronic bone diseases. Minor augmentative procedures to cover a few exposed implant threads were possible and not regarded as a contraindication. Prior to the enrolment into the investigation, the patients had to sign a written informed consent. The investigation was performed in compliance with the STROBE statement for strengthening the reporting of observational studies in epidemiology [27] and was in agreement with the appropriate EQUATOR guidelines [28].

### 2.3. Oral Implant Devices, Surgical and Prosthetic Procedures, Follow-Ups

An alumina-toughened zirconia (ATZ) implant (Metoxit AG, Thayngen, Switzerland) was used in this investigation. The enossal part of the implant showed a rough surface, which was produced according to a special additive surface technology. The implant surface was sandblasted as an initial procedure. This step presented a surface priming which was subsequently followed by the actual implant coating with the application of a ceramic slurry (mixture of a liquid and ceramic powder) onto the surface. While finally sintering the slurry on the implant, the liquid evaporated and led to a porous surface. The detailed pre- and operative steps have been described in a previous publication [25]. In brief, implants have been placed in healed ridge areas. After careful flap elevation, the implants were inserted in the prosthetically correct positions, minor augmentation procedures were performed if necessary, and the flaps closed back again. All implants were immediately temporized with relined eggshell temporaries. The occlusal as well as the approximal contacts were removed to reduce implant loading during oral function. For a standardized evaluation of the MBL over time, individual radiographic film holders were produced after implant placement. After 7 to 10 days, the sutures were removed, and the patients were placed on a biweekly control with professional cleaning. The temporaries were replaced through permanent reconstructions in the lower jaw not before 8 weeks and in the upper jaw not before 16 weeks of healing. SCs were fabricated out of IPS e.max CAD LT and the three-unit FDPs out IPS e.max ZirCAD veneered with IPS e.max ZirPress LT (both Ivoclar Vivadent, Schaan, Liechtenstein).

### 2.4. Clinical Peri-Implant Soft Tissue Evaluation

At prosthetic delivery, one and five years after implant installation, patient follow-ups were performed. At all follow-ups, clinical parameters (clinical attachment level (CAL), probing depth (PD), gingival/mucosal margin recession (GR), modified bleeding index (mBI), and modified plaque index (mPI) [29]) were recorded. The measurements were accomplished using a periodontal probe and the numbers for the CAL and PD rounded to the nearest millimeter.

The marginal bone loss development was evaluated from implant placement over one to five years with standardized radiographs. The magnification factor of the X-ray images was calibrated prior to the measurements on the basis of the thread distances (ImageJ, National Institutes of Health, Bethesda, MD, USA). The implant installation timepoint was set as baseline.

### 2.5. Patient-Reported Outcome Measures (PROMs; Patient’s Assessment)

The patients were asked to rate their satisfaction with the implant supported prosthetic reconstruction on a 10 cm visual analogue scale (VAS) regarding function, esthetics, whether the implants feel like their own teeth (sense), speech, and self-esteem/self-confidence. The assessment was performed before implant placement, at the delivery of the prosthetic reconstructions, and at the 1- and 5-year follow-ups. The patients could rate each parameter between 0% (poor) and 100% (excellent) with a perpendicular line on the VAS.

### 2.6. Implant Success Rating and Bone Loss Criteria

A successful implant was an implant that did not cause allergic, toxic, or gross infectious reactions either locally or systemically, which offered anchorage to a functional reconstruction and did not show any signs of radiolucency on intraoral radiographs. Implant fracture or mobility was regarded as failure.

The influence of the marginal bone loss on the implant success grading as suggested by Östman et al. [30] was adapted. Grade I success was applied for implants with no clinical and radiographic signs of pathology showing ≤2 mm marginal bone loss, and success grade II was assigned to all implants with ≤3 mm of marginal bone loss.

### 2.7. Statistical Analysis

For the statistical analyses, the software STATA 16.1 (StataCorp LT, College Station, TX, USA) using “xtmixed” was used. For the evaluation of the PROMs, linear mixed models with random intercepts were fitted for each participant to assess the effect of time and treatment (SC/FDP) on the assessment variables. To avoid a ceiling effect, we assumed a separate variance for each time point. For the peri-implant soft and bone tissue evaluation, linear mixed models with random intercepts were also fitted to evaluate the effect of time, location (implant or neighboring tooth), and type of reconstruction (SC/FDP) on the clinical/radiographical response parameters evaluated in the study (CAL, PD, GR, mBI, mPI/marginal bone loss). Since the data concerning the parameters included three positions (implant and two neighboring teeth), the patients were considered as clusters. The process of clustering was conducted individually for each of the above-mentioned variables. The method according to Scheffé was used to correct for multiple testing in pairwise comparisons (adjustment of *p*-values). The level of significance was set at *p* < 0.05. The statistical analysis was performed independently by a member (K.V.) of the Institute of Medical Biometry and Statistics, Faculty of Medicine and Medical Center—University of Freiburg.

## 3. Results

### 3.1. Status of Follow-Up

Initially, 27 patients received one implant for SC (27 implants), and 13 patients received two implants (26 implants) for a 3-unit FDP. Eighteen implants were located in the upper jaw. Seven implants had a diameter of 3 mm, 32 implants had a diameter of 4 mm, and 14 implants had a diameter of 5 mm. All implants showed an insertion torque of ≥30 Ncm. Three implants for single crowns were lost before prosthetic delivery. Hence, 50 implants were restored. Thirty-seven out of 40 patients were seen at the 1-year follow-up and 35 patients were seen at the 5-year follow-up. Between the 1-year and 5-year follow-up, one patient died and one patient moved to an unknown location (both with one implant each). After the 1-year follow-up, no further implant loss occurred, which led to a cumulative implant survival rate of 94.3% after 5 years (Table 1).

### 3.2. Marginal Bone Loss

**Figure 1:**
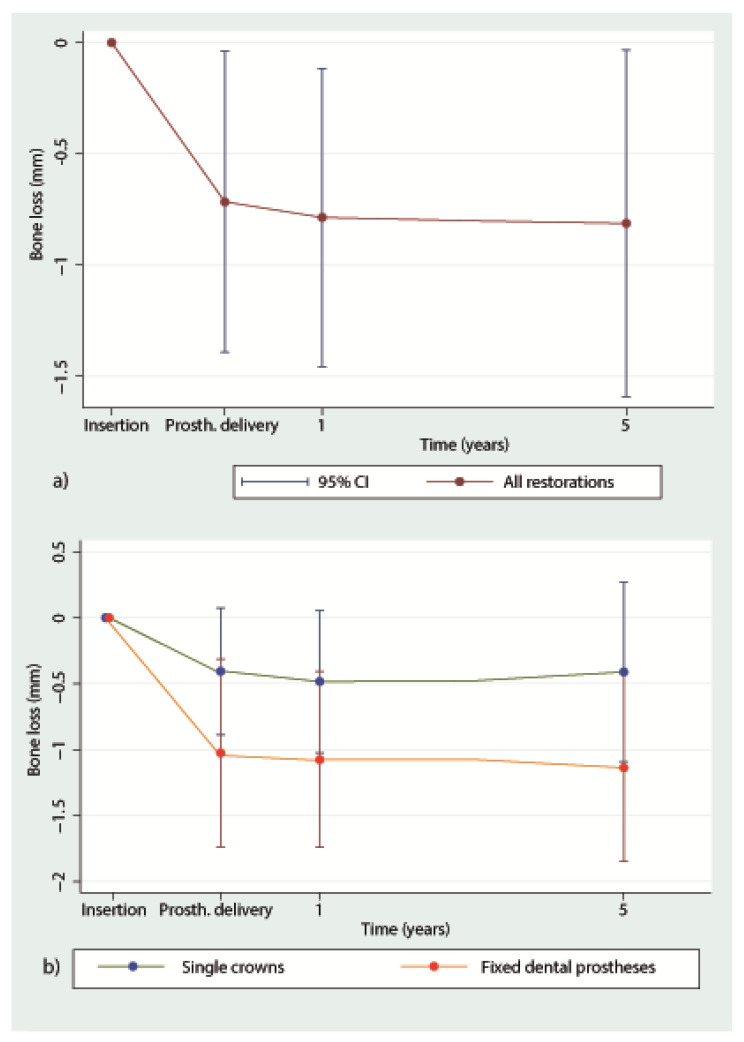
(**a**). Mean marginal bone loss (± Standard Deviation) irrespective of reconstruction from implant insertion until the 5-year follow-up. (**b**) Mean marginal bone loss for single crowns (SC) and fixed dental prosthesis (FDP) from implant insertion to the 5-year follow-up. 1 = 1-year follow-up; 5 = 5-year follow-up.

**Figure 2:**
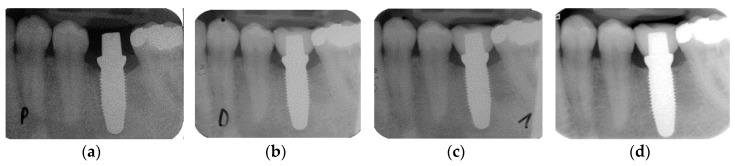
Standardized radiographs for the assessment of marginal bone loss (MBL): (**a**) implant insertion; (**b**) prosthetic delivery; (**c**) one-year follow-up; (**d**) five-year follow-up.

The marginal bone loss from implant installation to the 5-year follow-up for all implants is depicted in Figure 1a. The bone loss from implant insertion until the reconstruction delivery was 0.71 mm (± 0.67), 0.78 mm (± 0.67) at the 1-year follow-up and 0.81 mm (± 0.77) at the 5-year recall. The bone loss from implant installation to the delivery was statistically significant (*p* < 0.001), while the loss from the prosthetic delivery to the 1-year (*p* = 0.548) and to the 5-year follow-up (*p* = 0.611) was not (Table 2). Four implants showed bone loss >2 mm at 5 years. Two implants had already an increased bone of >2 mm loss at 1 year, and their situation was stable up to 5 years. The other two implants showed an increase in radiographically measured bone loss of approximately 1 mm over the time interval of 4 years. Conventional maintenance on a regular basis stabilized the situation. From implant insertion to the 5-year follow-up, five implants (10.4%) gained bone, whereas four implants (8.3%) lost > 2 mm (Table 3). None of the implants lost more than 3 mm of bone. Hence, when applying the success and failure criteria according to Östman et al. [30], 91.7% of the remaining implants were successful according to criteria I and 100% were successful according to criteria II. A bone loss difference was observed between implants used for SC and implants used for FDP up to the 5-year follow-up (Figure 1b). The bone loss at prosthetic delivery was 1.03 mm (±0.71) for the FDPs and 0.41 mm (±0.48) for the SCs (*p* = 0.001). At the 1-year follow-up, the marginal bone reduction was 1.08 mm (±0.67) for the FDPs and 0.48 mm (±0.54) for the SCs (*p* = 0.001). At the 5-year follow-up, the FDPs showed a bone loss of 1.14 mm (±0.71) and the SCs showed a bone loss of 0.41 mm (±0.68) (*p* = 0.001). In the univariate analysis (Table 4) of the baseline variables gender, jaw, position in the jaw, implant diameter and length, bone quality and quantity, anchorage, grafting and flap design, no significant influence on bone loss could be found. Figure 2 presents exemplary follow-up radiographs of an SC. 

### 3.3. Peri-Implant Soft Tissue Evaluation (Table 5)

Probing depth: The probing depth at the mesial abutment tooth was 2.2 mm at prosthetic delivery and increased to 2.6 mm at the 5-year follow-up (*p* = 0.005). At the implant sites, the PD increased from 2.7 mm at the delivery of the restorations to 3.3 mm at the 5-year follow-up (*p* < 0.001). The change in PD at the distal teeth was not significant (delivery and 5-year follow-up: both 2.4 mm; *p* = 0.787).

Clinical attachment level: The change of the CAL from prosthetic delivery to the 5-year follow-up was not significant neither at teeth nor implant sites (implant sites, prosthetic delivery: 2.6 mm, 5-year follow-up: 2.7 mm) (all *p* > 0.05).

Gingival/mucosal margin recession: The soft tissue margin around teeth as well as around implants was stable. There was a slight increase in recession around the mesial tooth (delivery—5-year follow-up: 0.08 mm), which was not significant (*p* = 0.127). A decrease of GR occurred at implant (0.09 mm; *p* = 0.005) and distal tooth sites (0.08 mm; *p* = 0.955).

Modified Plaque Index: The plaque index (mPI) increased statistically non-significant at mesial tooth sites (0.52 to 0.61; *p* = 0.277), but it was statistically significant at implant sites (0.29 to 0.49; *p* = 0.000). At distal tooth sites, the index showed a reduction from 0.76 to 0.69 (*p* = 0.002).

Modified Bleeding Index: The bleeding index (mBI) decreased around the teeth (mesial: from 0.36 to 0.20; *p* < 0.001; distal: from 0.43 to 0.38; *p* = 0.708). The mBI increased significantly at implant sites from delivery to the 5-year follow-up (from 0.51 to 0.87; *p* < 0.001).

### 3.4. Patient Assessment: Patient-Reported Outcome Measures

**Figure 3:**
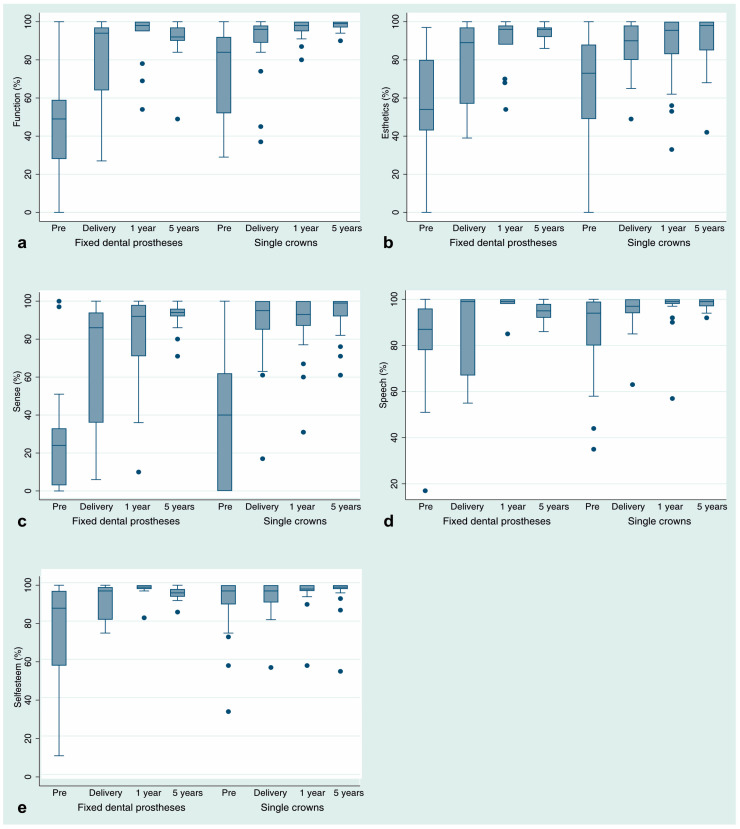
Box plot diagrams of patient-reported outcome measures (visual analog scales, %; (**a**).: function (eating); (**b**).: esthetic/appearance; (**c**).: sense; (**d**).: speech; (**e**).: self-esteem) sorted by restoration type (fixed dental prostheses, single crowns) before treatment (p pre), at prosthetic delivery (delivery), and at the follow-up appointments (1-y follow-up; 5-y follow-up). The lower horizontal lines represent the minimal documented values and the highest ones the maximal values. The lowest end of the box represents quartile 1 and the highest quartile 3. The bold line within the box is the median. The dots represent outliers.

The PROMs results are presented in Figure 3 and Table 6. Compared with the pretreatment situation (33.9–85.2%), all assessments revealed significantly improved average VAS values at the delivery of the prosthetic restorations (81% to 93.5%). Whereas the improvement of function, speech, and self-esteem remained stable over the course of the follow-ups, subjective patients’ perceptions of esthetics and sense still significantly increased over time.

## 4. Discussion

Mid- and long-term results are rare for zirconia ceramic implants [6,7,8]. Therefore, the present 5-year results of an alumina-toughened zirconia implant adds to the information on zirconia implants and confirms the initial positive clinical and radiographic results of the shorter-term outcomes of this type of implant system [25,26]. Three implants were lost in total, all prior to prosthetic delivery. Reasons for early implant failure might have been attributable to the developmental phase of the investigation [31] and to a certain learning curve of the operators. However, it also might have been a coincidence that the three first implants have been lost. The null hypothesis assuming that there is no difference in terms of implant survival rates between one (94.3%) and five (94.3%) years after implant placement could not be rejected. The ATZ-implant performed well over the entire follow-up period of five years. The estimated survival rate of zirconia implants was indicated in meta-analysis with 91% [2] to 96% [3] at one year. When focusing only on commercially available implants, the estimated survival rate increased to 98% [2]. Based on follow-up times exceeding one year, an expected decrease of implant survival of 0.05% per year was calculated [3]. The estimated 2-year survival rate for commercially available implants was calculated with 97% [2]. The present 5-year data on implant survival coincide well with the 5-year survival found for SC and FDP on one- [32] and two-piece titanium implants [33,34].

Regarding implant success on the basis of “marginal bone loss”, the success grade I at 5 years (91.7%) decreased compared to the one-year follow-up. Four implants showed an increased bone loss between 2 and 3 mm at 5 years (8.3%); two of these implants already experienced this bone loss after the first year. Similar findings regarding the bone loss surrounding two-piece titanium implants after one year was found by Östman and coworkers [30]. These observations seem to be comparable to the outcome of standard “two-piece titanium implants” [33,34] and are therefore encouraging.

A statistically significant marginal bone loss in the present investigation was detected from the timepoint of implant placement until the delivery of the prosthetic reconstruction. A significant initial bone loss was also shown in other investigations applying one-piece zirconia implants [35,36]. Therefore, this “remodeling” seems to be the result of the surgical intervention with the subsequent healing and may be regarded as a biological reaction toward the trauma. No further significant bone loss was observed thereafter. These results are not different to one- or two-piece titanium implants [32,37]. In 10.4% of the implants, a marginal bone gain was observed, which might be related to the osteoconductive surface of this implant type counteracting bone loss after healing.

Significant higher MBL resulted for implants supporting FDPs compared to SCs, especially before delivery of the definitive reconstructions. This might have been caused by a higher load transmission to the provisional bridges, which were in general not protected by teeth distally to the implants. In SC reconstructions, the embedding of the implants in between two teeth could have possibly protected the implant from masticatory forces and from tongue and cheek pressure, which might have led to lesser bone loss in comparison to FDPs.

Regarding the peri-implant and periodontal soft tissues, slight changes in values over the observational period of 5 years were observed. Probing depths values around implants were higher than around teeth [8]. This difference seems to be a normal and frequent finding, since animal investigations were able to show that the peri-implant soft tissue does not resist probing to the same extent that periodontal tissue does. The probe tip was found closer to the bone in peri-implant tissues than around periodontal tissues [38,39]. Probing depth values around titanium implants were also found to be higher than around natural teeth [40] at 1 year [41] and 5 years [42]. Therefore, this seems to be a common outcome and might not be related to implant material or design.

Although the Plaque Index at five years was significantly lower at implants compared to teeth, the Bleeding Index was significantly higher. The increased Bleeding Index around implants might be the results of the probe tip penetrating deeper into the sulcus around implants than around teeth and therefore damaging more blood vessels [43]. A higher bleeding tendency around implants compared to teeth is also a common finding in the implant literature [8,40].

From prosthetic delivery to the 5-year follow-up, there was an increase of the mPI from 0.29 to 0.49 at the implant sites. Although this increase was statistically significant, the oral hygiene of the patient cohort nevertheless was regarded as good at the 5-year follow-up. The increase of the mPI was attributable to the crown design. Since the ATZ implants were one-piece implants, they were placed with the implant–crown margin slightly below the soft tissue, where possible. In turn, this had the consequence that the crowns presented an emergence profile with a flat angle in relation to the soft tissues, rendering the cleaning of the crowns more difficult.

The different healing periods for the upper and lower jaw were based on information from studies that showed that the upper jaw had a lower bone mineral density (i.e., bone quality) than the lower jaw [44,45]. Higher failure rates were reported for lower bone qualities [46,47]. At the timepoint of the study design development, no clinical data were available on the integration of the ATZ implants in different bone quality types. For reasons of patients’ safety, it was decided to use an increased implant healing period before definitive reconstruction for the upper jaw in comparison to the lower jaw. An aspect that is actually not directly related to the implant material or implant design but to implant treatment per se is patient-reported outcome measures (PROMs). PROMs aim to assess people’s subjective perception of health, addressing their satisfaction with the treatment and their oral health related quality of life. In our investigation, we were able to follow the satisfaction development of the participating patients over 5 years. Before treatment, the patients were less satisfied with the overall situation of their reduced dentition (in some areas below 70% of satisfaction) compared with after the treatment. With the delivery of the restorations, all PROMs aspects (function, esthetics, sense, speech, and self-esteem) increased to more than 80% for the first three parameters and to more than 90% for the latter two. At the 1-year follow-up, esthetics and sense were well above 80% and function, speech, and self-esteem were well above 90%. Even at the 5-year follow-up, there was still an increase for all aspects, and esthetics and sense showed a significant improvement to the results at the 1-year follow-up. The delivered treatment “zirconia implants and prosthetic reconstructions” had a positive effect on patient satisfaction and can be regarded as successful from a patient’s perspective over a mid-term period.

This investigation was a prospective observational study. No titanium control group as in a randomized controlled investigation was used. This has to be regarded as a drawback, since regarding the level of evidence, the latter one is superior to the former design. Furthermore, the cohort group of 40 patients might be considered small. However, with respect to the other investigations presented in a systematic review [2], where the number of patients is indicated between 12 and 74, our cohort group with 40 patients is in between these numbers. Nevertheless, the present type of investigation may add to the knowledge of zirconia implants. We were able to show that the results from the application of this type of implant over 5 years are acceptable to good regarding the survival, radiographic, and clinical performance. Patients were very satisfied with the treatment results for over a period of 5 years.

## 5. Conclusions

Within the limitations of the present investigation, the evaluated implant can be considered to be safe and reliable for the reconstruction of implant-supported SCs and FDPs over a mid-term period. The present findings substantiate the clinical applicability of this system.

## Figures and Tables

**Table 1:** Status of follow-up.

Time	N of Implants	Losses	Survival (%)	Survival Worst-Case Scenario (%)
Baseline to delivery	53	3	94.3	94.3
at delivery	50	0	94.3	94.3
Delivery to 1-year follow-up	50	0	94.3	94.3
1-year to 5-year follow-up	48	0 (2 *)	94.3	90 *

* Survival when the implants of the dropped-out patients are counted as losses.

**Table 2:** Marginal bone loss at prosthetic delivery and at the 1- and 5-year follow-up (mesial reference tooth, implant site, distal reference tooth).

	Prosthetic Delivery			1-y Follow-Up			5-y Follow-Up					Significance (*p*)			
	*n*	Mean	SD	*n*	Mean	SD	*n*	Mean	SD	Mean	SD	I → D	D → 1y	D → 5y	1y → 5y
Position															
Mesial tooth	30	0.06	0.92	35	0.11	0.63	32	0.39	1.50	0.39	1.50	0.593	0.523	0.294	0.292
Implant	45	0.71	0.67	48	0.78	0.67	48	0.81	0.78	0.81	0.77	<0.001 *	0.548 *	0.611 *	0.892 *
Distal tooth	24	0.12	0.64	25	0.35	0.58	23	0.41	0.90	0.41	0.89	0.170	0.029	0.151	0.734

The marginal bone level at the day of implant insertion (I) served as baseline. Significant differences regarding the reconstruction type (single crowns or fixed dental prosthesis) were superscripted with *. *n* = number of observations; SD = Standard Deviation; I = Implant insertion; D = Prosthetic delivery; 1y = 1-year follow-up; 5y = 5-year follow-up.

**Table 3:** Frequency analysis of marginal bone loss at prosthetic delivery and at the 5-year follow-up.

	Insertion to Prosthetic Delivery		Insertion to 5-Year Follow-Up	
**Bone loss in mm**	***n***	**%**	***n***	**%**
<0	7	15.6	5	10.4
0	1	2.2	0	0
>0 –0.5	9	20	12	25
>0.5–1.0	14	31.1	11	22.9
>1.0–1.5	10	22.2	11	22.9
>1.5–2.0	2	4.4	5	10.4
>2.0–2.5	2	4.4	3	6.25
>2.5–3.0	0	0	1	2.1
Total	45	100	48	100

**Table 4:** Univariate analysis of marginal bone loss from implant insertion to the 5-year follow-up (n.e.o.: not enough observations).

Variable	*n*	Mean (in mm)	Standard Deviation	*p*-Value
**Gender**				
female	23	0.87	0.78	0.645
male	25	0.76	0.78	
**Jaw type**				
mandible	32	0.78	0.79	0.680
maxilla	15	0.89	0.78	
**Ant-post**				
posterior implants	44	0.81	0.79	0.855
anterior implants	3	0.87	0.63	
**Implant diameter in mm**				
3	6	1.05	0.57	0.205
4	29	0.89	0.83	
5	12	0.50	0.71	
**Implant length in mm**				
9	15	1.14	0.78	0.071
12	28	0.72	0.71	
14	4	0.25	0.93	
**Bone quality**				
1 and 2	36	0.78	0.77	0.207
3	11	1.05	0.79	
**Bone quantity**				
A	20	0.65	0.79	0.245
B and C	27	0.93	0.76	
**Anchorage**				
no cortical	2	0.60	0.57	n.e.o.
monocortical	42	0.80	0.81	
bicortical	3	1.07	0.60	
**Grafting**				
no	22	0.82	0.66	0.987
yes	25	0.75	0.81	
**Flap design**				
w/o releasing incisions	39	0.86	0.80	0.477
w releasing incisions	8	0.65	0.60	
**Torque**				
> 30 ≤ 35 Ncm	32	0.94	0.78	0.064
> 35 Ncm	15	0.55	0.72	

**Table 5:** Soft tissue evaluation at prosthetic delivery and at the 5-year follow-up.

	Prosthetic Delivery			5-y Follow-Up			Significance (*p*)
	*n*	Mean	SD	*n*	Mean	SD	D → 5y
**PD in mm**							
Mesial tooth	35	2.19a	0.74	35	2.56a	1.41	0.005
Implant	47	2.67b	0.75	48	3.27b	0.64	<0.001 *
Distal tooth	26	2.38b	0.63	24	2.44a	0.64	0.787
**CAL in mm**							
Mesial tooth	35	2.68ab	1.00	35	2.8a	0.85	0.293
Implant	47	2.64a	0.92	48	2.68b	0.80	0.986
Distal tooth	26	2.74b	0.84	24	2.59a	0.98	0.142
**GR in mm**							
Mesial tooth	35	0.44a	0.63	35	0.52a	0.54	0.127
Implant	47	0.34a	0.43	48	0.25b	0.30	0.005
Distal tooth	26	0.54a	0.59	24	0.46ab	0.57	0.955
**mPI**							
Mesial tooth	35	0.52a	0.60	35	0.61a	0.52	0.277
Implant	47	0.29b	0.41	48	0.49b	0.47	<0.001 *
Distal tooth	26	0.76c	0.61	24	0.69a	0.58	0.022
**mBI**							
Mesial tooth	35	0.36a	0.39	35	0.20a	0.29	<0.001
Implant	47	0.51a	0.41	48	0.87b	0.54	<0.001 *
Distal tooth	26	0.43a	0.42	24	0.38c	0.55	0.708

*n* = number of units, SD = standard deviation, PD = probing depth, CAL = clinical attachment level, GR = “gingival” recession, mPI = modified Plaque Index, mBI = modified Bleeding Index. Different bold letters (a, b, c) behind the mean values indicate significant differences between teeth and implants. Significant differences regarding the type of reconstruction (SC or FDP) were superscripted with *. D = Prosthetic delivery; 5y = 5-year follow-up.

**Table 6:** Patient’s assessment of function, esthetics, sense, speech and self-esteem using visual analogue scales (VAS in %) at pretreatment, prosthetic delivery, and at 1- and 5-year follow-ups. Significances were calculated for changes between Pretreatment (Pre) and prosthetic delivery (D) and from Delivery to the 1-year follow-up and from the 1-year follow-up to the 5-year follow-up. SD = Standard Deviation.

Assessment Parameter		Pre	D	1-Year Follow-Up	5-Year Follow-Up	Significance (*p*)		
						Pre → D	D →1 year	1 year → 5 years
**Function**	*n*	40	34	37	35			
	Mean (%)	65.3	86	94.7	95	<0.0001	0.001	0.220
	SD	27.8	19.7	9.8	9.1			
**Esthetics**	*n*	40	34	37	35			
	Mean (%)	61.5	84.8	87.7	91.9	<0.0001	0.313	0.012
	SD	29.5	16.5	17.1	12.2			
**Sense**	*n*	40	34	37	35			
	Mean (%)	33.9	81	83.8	92.6	<0.0001	0.606	0.001
	SD	33.8	26.7	23.5	10.1			
**Speech**	*n*	40	34	37	35			
	Mean (%)	85.1	92.7	96.9	96.8	0.026	0.020	0.689
	SD	19.5	12.3	7.5	3.5			
**Self-esteem**	*n*	40	34	37	35			
	Mean (%)	85.2	93.5	96.8	95.9	0.032	0.004	0.491
	SD	23.1	9.2	7.3	8			
